# Red cell distribution width to albumin ratio is a risk factor for atrial fibrillation in subjects hospitalized with coronary angiography

**DOI:** 10.1186/s12872-024-03772-8

**Published:** 2024-02-08

**Authors:** Wenhua Li, Yanbin Song

**Affiliations:** 1 Department of Cardiology, Wujin Hospital Affiliated With Jiangsu University, Changzhou, 213017 China; 2 Department of Cardiology, the Wujin Clinical College of Xuzhou Medical University, Changzhou, 213017 China

**Keywords:** Red cell distribution width, Albumin, Atrial fibrillation

## Abstract

**Background:**

Red cell distribution width to albumin ratio (RAR) has been demonstrated to be associated with the risk of cardiovascular diseases. However, it is still unknown whether the RAR affects atrial fibrillation (AF). Therefore, this study aimed to investigate the association between RAR and AF in subjects hospitalized with coronary angiography.

**Methods:**

A total of 2436 participants were retrospectively included. Red cell distribution width, albumin and other data were collected. AF was confirmed using 12-lead electrocardiogram (ECG) or 24-h Holter. All participants were divided into four groups according to the RAR values by quartile (Q1, Q2, Q3, Q4). Univariate and multivariate logistic regression were performed to examine the correlation between RAR and AF.

**Results:**

Among the 2436 participants, 227 (9.3%) AF cases were observed. The RDW and RAR were significantly higher in AF group than in non-AF group (all *P* < 0.001). Univariate logistic regression showed an positive association between RAR and AF (*P* < 0.001). In multivariate logistic regression, RAR was found to be an independent risk factor of AF after adjusting for confounding factors (OR:2.015, 95%CI:1.315–3.089, *P* = 0.001).

**Conclusions:**

The present study indicated that elevated RAR level was independently correlated with increased risk of AF in subjects hospitalized with coronary angiography.

**Supplementary Information:**

The online version contains supplementary material available at 10.1186/s12872-024-03772-8.

## Background

Atrial fibrillation (AF) is the most common cardiac arrhythmia in clinical practice for decades, the prevalence of AF ranges from 2 to 12% in the general population [1, 2]. Epidemiology studies have shown that AF is associated with acute myocardial infarction, heart failure, stroke, death, peripheral embolism, and other adverse outcomes [2, 3]. Up to now, fibrosis, inflammation and oxidative stress are reported to influence many cardiovascular diseases. Moreover, recent study have found that novel ECG parameters P-wave peak time in V1 lead (PWTV1) and P-wave peak time in D2 lead (PWPTD2) associated with atrial remodeling have high prognostic value in predicting patients likely to develop AF. And the ATRIA stroke risk scoring system performs better than other commonly used risk scoring systems in predicting atrial high-rate episodes correlated with AF [4, 5]. However, the mechanisms of developing and maintaining AF remain unclear [6]. Although clinical treatments of AF have been developed to reduce the burden of disease, the estimated number of patients in the world is up to 33.5 million [7]. Therefore, it is important to identify the modifiable risk factors for AF.

Previous studies have demonstrated that the red cell distribution width (RDW), an index of variation of erythrocyte volume, is associated with cardiovascular death, heart failure, fatal coronary disease and sub-clinical inflammation [8–10]. Low serum albumin levels are also linked to ischemic heart disease, heart failure, atrial fibrillation, stroke and inflammation [11]. Albumin affected by chronic inflammation has great value when assessing long-term mortality in patients with permanent pacemakers [12]. Clinical evidence indicates that low albumin appears to be independently associated with a long-term risk of AF in octogenarians after having dual chamber permanent pacemaker implanted [13]. In additional, a novel inflammatory marker, the uric acid/albumin ratio (UAR), was found to be correlated independently with AF recurrence after catheter ablation [14].

The RAR, a combination of red cell distribution width and albumin, is a novel index of inflammation. Prior researches have suggested that RAR may be helpful in assessing the outcomes in of acute biliary pancreatitis attacks [15]. 60-day mortality in patients with acute respiratory distress syndrome [16]. and carotid plaques in patients with CHD [17]. However, the association of RAR and AF has not been clarified in prior studies. The current study was therefore carried out to investigate the association among subjects hospitalized with coronary angiography in our center.

## Methods

### Study Participants

For the cross-sectional study, a total of 2584 consecutive participants who had been admitted to our center due to chest pain or tightness and underwent coronary artery angiography (CAG) for the first time between 2016 and 2020 were retrospectively enrolled.

**Fig. 1:**
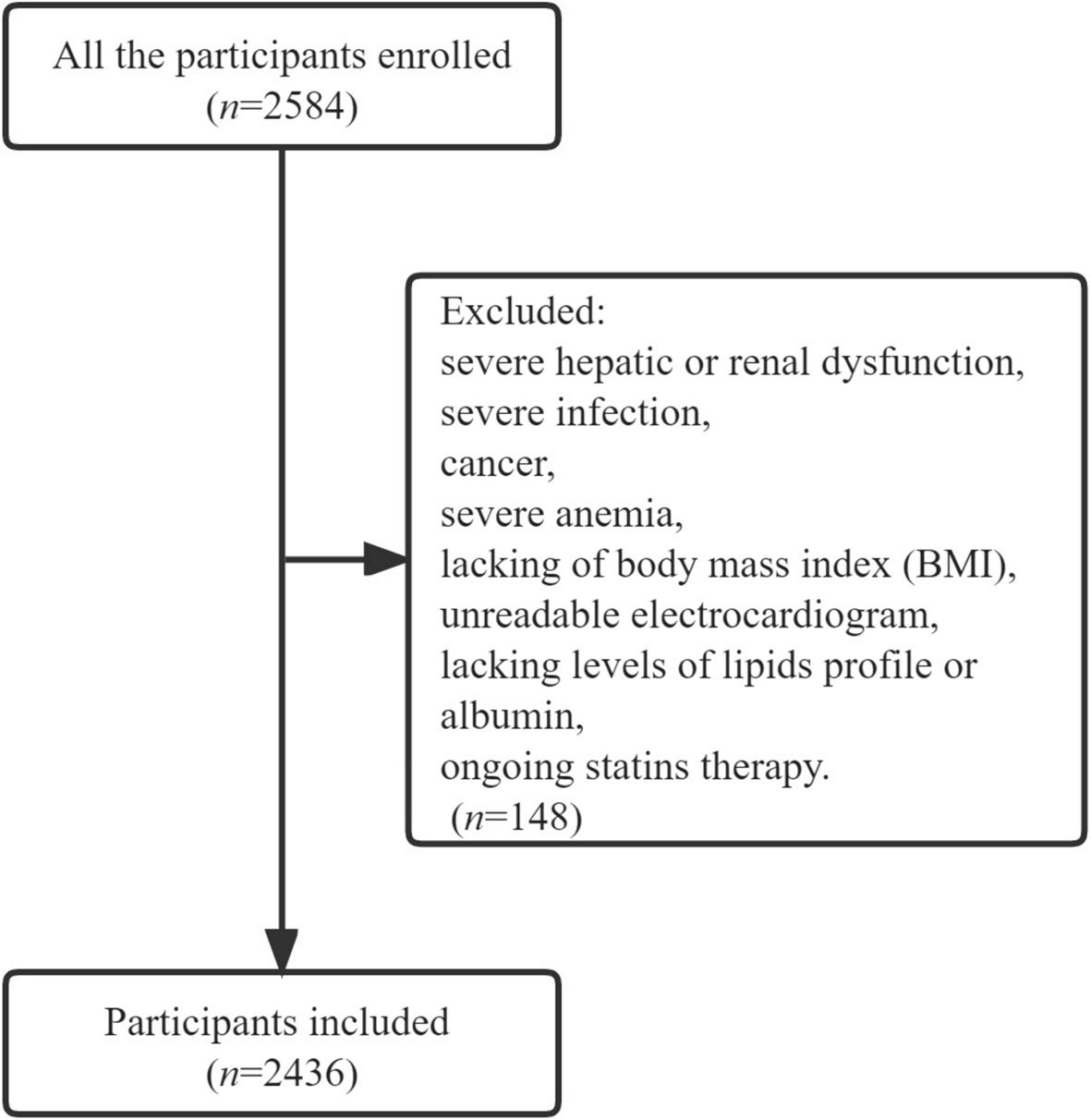
The flowchart of selecting participants for analysis

The inclusion criteria for the study included adults ≥ 18 years old who had essential electrocardiogram and complete sets of data. Exclusion criteria included severe hepatic or renal dysfunction, severe infection, cancer, severe anemia, unreadable electrocardiogram, lacking of body mass index (BMI), or levels of lipids profile or albumin, ongoing statin therapy. Finally, 2436 eligible participants were included in the data analysis (Fig. 1).

This study was carried out in accordance with the ethical guidelines of the Declaration of Helsinki. The written informed consents for participation were not presented owing to the retrospective nature of this study. Additionally, this study was approved by the Ethics Committee of Wujin Hospital Affiliated with Jiangsu University, which waived the need for informed consent.

### Data Collection

The data were collected from the electronic medical records. Demographic and clinical data, including age, sex, BMI, smoking, drinking, hypertension, diabetes mellitus (DM), and coronary heart disease (CHD), as well as the medication (Beta-blockers) were collected by cardiologists. Fasting blood samples were collected either before CAG or early the next morning after emergency CAG. The biochemical parameters including fasting serum total cholesterol (TC), triglyceride (TG), low-density lipoprotein cholesterol (LDL-C), high-density lipoprotein cholesterol (HDL-C), red cell distribution width (RDW), albumin, white blood cell (WBC) and neutrophil for all included patients were measured using standardized laboratory methods (Supplement Table 1). RAR was calculated as follows: red cell distribution width / albumin. Level of RAR was divided into four groups by quartile.

**Table 1:** Characteristics of the included participants at baseline

Variables	AF (*n* = 227)	Non-AF (*n* = 2209)	*P* value
Age (y)	70(63,76)	65(56,71)	< 0.001
Male (n, %)	130(57.3)	1420(64.3)	0.036
Hypertension (n, %)	160(70.5)	1464(66.3)	0.200
DM (n, %)	45(19.8)	518(23.4)	0.217
CHD (n, %)	107(47.6)	1623(73.5)	< 0.001
Smoking (n, %)	66(29.1)	850(38.5)	0.005
Drinking (n, %)	36(15.2)	317(14.4)	0.539
BMI (kg/m^2^)	24.77(22.49,27.22)	24.53(22.43,26.75)	0.111
Beta-blockers (n, %)	31(13.7)	249(11.3)	0.337
WBC (×10^9^/L)	6.25(5.38,8.06)	6.75(5.44,8.37)	0.025
Neutrophil (×10^9^/L)	4.07(3.12,5.42)	4.23(3.25,5.77)	0.076
TC (mmol/L)	4.15(3.41,4.75)	4.43(3.74,5.13)	< 0.001
TG (mmol/L)	1.37(0.95,1.93)	1.55(1.10,2.26)	< 0.001
HDL-C (mmol/L)	1.07(0.92,1.24)	1.07(0.93,1.26)	0.688
LDL-C (mmol/L)	2.57(2.07,3.17)	2.85(2.28,3.43)	< 0.001
RDW (%)	12.70(12.20,13.20)	12.50(12.20,13.30)	< 0.001
Albumin (g/dL)	38.30(35.60,40.70)	39.10(36.90,41.50)	< 0.001
RAR (%/g/dL)	3.35(3.13,3.63)	3.19(2.98,3.44)	< 0.001

*DM *Diabetes mellitus, *CHD* Coronary heart disease, *BMI* Body mass index, *WBC* White blood cell, *TC* Total cholesterol, *TG* Triglyceride, *LDL-C* Low-density lipoprotein cholesterol, *HDL-C* High-density lipoprotein cholesterol, *RDW *Red cell distribution width, *RAR* Red cell distribution width/albumin ratio

### Definitions

The AF was confirmed using 12-lead electrocardiogram (ECG) or 24-h Holter. AF rhythm was defined as (I) irregular R-R intervals (II) absence of distinct repeating P waves (III) irregular atrial activity show on ECG [18]. The final diagnosis of AF were reviewed and conformed by both the researchers.

Hypertension was defined as a systolic blood pressure (SBP) of ≥ 140 mmHg and/or a diastolic blood pressure (DBP) of ≥ 90 mmHg or with the use of any anti-hypertensive drug [19].

Diabetes mellitus (DM) was diagnosed based on FBG ≥ 7.0 mmol/l or 2-h post-load glucose ≥ 11.1 mmol/l or current use of anti-diabetic medications [20].

Coronary heart disease (CHD) was diagnosed as stenosis of 50% or more of the diameter of the major coronary blood vessels [21].

### Statistical Analysis

Statistical analysis was performed using SPSS version 25.0. The normally distributed continuous variables were expressed as mean ± standard deviations. And the non-normally distributed continuous variables were expressed as median (first and third quartile). The comparisons in continuous variables were analyzed by the Student *t* test, One-Way ANOVA or non-parametric Mann–Whitney test, as appropriate. Categorical variables were described as frequencies and percentages. Differences between groups were tested with the Chi-square for categorical variables. Univariate logistic regression analysis was used to identify the risk factors for AF. Meanwhile, multivariate logistic regression analysis was carried out to evaluate the association between RAR and AF after adjusting for potential confounding variables. Potential confounding factors adjusted for in multivariable analysis were determined based on previous studies and the univariate logistic regression analysis. Odds ratio (OR) and 95% confidence interval (95% CI) were presented for the logistic regression analyses. A receiver operating characteristics (ROC) curve was constructed to assess the predictive value of RAR for AF. All* P* values were two sided. A *P* value less than 0.05 was considered as statistically significant.

## Results

### Baseline Characteristics of the Study Population

In our study, 98.68% (224/227) of patients with AF had been confirmed before CAG. Only three acute myocardial infarction (AMI) patients with paroxysmal atrial fibrillation was diagnosed after emergency CAG.

The baseline characteristics for the study participants were summarized in Table 1. The median age of the study participants was 65 years, and 1550 (63.6%) were males. A total of 227 (9.3%) AF patients were identified. Patients with AF were older, had higher proportion of male gender and smoking status (*P* < 0.05). The presence of AF was strongly inversely associated with CHD diagnosed on CAG (*P* < 0.001). Compared to those without AF, patients with AF had significantly lower levels of TC, TG, LDL-C and albumin (all *P* < 0.001). Individuals with AF were more likely to had lower levels of WBC (*P* = 0.025). The RDW and RAR levels were significantly higher in patients with AF than in subjects without AF (all *P* < 0.001).

There was no significant difference in the proportion of HBP, DM, drinking history between AF and non-AF group in over population. The statistically significant difference was also not observed in levels of BMI and HDL-C between the two groups.

### Comparison of RAR quartile

Then, the participants were assigned into four groups (Q1: < 2.99, Q2:2.99–3.20, Q3: 3.20–3.45, Q4: ≥ 3.45) according to the RAR quartile. As shown in Table 2, patients with the highest RAR level tended to be older ( 69 versus 62 years), had a higher proportion of AF (14.6% versus 6.3%), lower levels of TC, TG, LDL-C and albumin compared to those with lowest RAC level (all* P* < 0.001). In addition, higher WBC and neutrophil levels occurred more frequently in the higher RAR group. Furthermore, participants in Q4 group were likely to have higher RDW levels than those in Q1 group (* P* < 0.001).

**Table 2:** Baseline characteristics of study participants by RAR quartile

Variables	Q1 (*n* = 608)	Q2 (*n* = 610)	Q3 (*n* = 609)	Q4 (*n* = 609)	*P* value
Age (y)	62 (54, 68)	65(55,71)	67(59,73)	69(61,74)	< 0.001
Male (n, %)	377 (62.0)	382 (62.6)	391 (64.2)	400 (65.7)	0.541
Hypertension (n, %)	406 (66.8)	414 (67.9)	419 (68.8)	385 (63.2)	0.179
DM (n, %)	152 (25.0)	146 (23.9)	134 (22.0)	131 (21.5)	0.431
CHD (n, %)	403 (66.3)	435 (71.4)	431 (71.0)	461 (75.8)	0.004
AF (n, %)	38 (6.3)	34 (5.6)	66 (10.8)	89 (14.6)	< 0.001
Smoking (n, %)	211 (34.7)	235 (38.5)	225 (36.9)	245 (40.2)	0.231
Drinking (n, %)	89 (14.6)	86 (14.1)	84 (13.8)	94 (15.4)	0.858
BMI (kg/m^2^)	24.80 (22.84,27.04)	24.90(22.86,26.74)	24.39(22.45,26.78)	24.03(21.64,26.56)	< 0.001
WBC (×10^9^/L)	6.49(5.36,7.86)	6.66(5.39,8.18)	6.82(5.42,8.61)	6.89(5.53,8.89)	0.022
Neutrophil (×10^9^/L)	4.02(3.12,5.26)	4.10(3.25,5.55)	4.27(3.25,6.15)	4.42(3.37,6.31)	< 0.001
TC (mmol/L)	4.58(3.88,5.33)	4.48(3.79,5.14)	4.39(3.70,5.10)	4.11(3.49,4.82)	< 0.001
TG (mmol/L)	1.68(1.22,2.54)	1.63(1.17,2.27)	1.52(1.11,2.21)	1.29(0.92,1.88)	< 0.001
HDL-C (mmol/L)	1.11(0.96,1.29)	1.08(0.93,1.27)	1.06(0.94,1.26)	1.04(0.90,1.20)	0.769
LDL-C (mmol/L)	2.93(2.33,3.49)	2.90(2.33,3.42)	2.84(2.24,3.47)	2.60(2.16,3.22)	< 0.001
RDW (%)	12.05(11.80,12.40)	12.3(12.0,12.7)	12.6(12.3,13.1)	13.2(12.65,13.70)	< 0.001
Albumin (g/dL)	43.0(41.4,44.7)	39.9(38.6,41.0)	38.0(36.9,39.25)	35.3(33.6,36.8)	< 0.001

*DM *Diabetes mellitus, *CHD* Coronary heart disease, *BMI* Body mass index, *WBC* White blood cell, *TC* Total cholesterol, *TG* Triglyceride, *LDL-C* Low-density lipoprotein cholesterol, *HDL-C* High-density lipoprotein cholesterol, *RDW* Red cell distribution width, *RAR* Red cell distribution width/albumin ratio

### Association of RAR and AF

The results of logistic regression analyses were presented in Table 3. Univariate logistic regression showed that age (OR:1.056, 95% CI:1.040–1.071,* P* < 0.001) and RDW (OR:1.414, 95% CI:1.218–1.642,* P* < 0.001) were significantly associated with increase risk of AF. Male gender (OR:0.745, 95%CI:0.564–0.982, *P* = 0.037), CHD (OR:0.322, 95% CI:0.244–0.425, *P* < 0.001), smoking (OR:0.655, 95% CI:0.0.486–0.884, *P* = 0.006), TC (OR: 0.728, 95% CI:0.636–0.834, *P* < 0.001), TG (OR:0.812, 95% CI:0.709–0.931, *P* = 0.003), LDL-C (OR:0.724 95% CI:0.615–0.852, *P* < 0.001), albumin (OR:0.936, 95% CI:0.902–0.971, *P* < 0.001), WBC (OR:0.931, 95% CI:0.880–0.986, *P* = 0.014), and neutrophil (OR:0.940, 95% CI:0.885–0.998, *P* = 0.042) were all statistically associated with a low risk of AF. And a positive relationship was observed between the RAR and AF risk (OR:2.567, 95% CI:1.724–3.822, *P* < 0.001).

**Table 3:** Univariate and multivariate logistic regression for the relationship between variables and AF

Variables		Univariate			Multivariate	
	OR	95% CI	***P***	OR	95% CI	***P***
Age	1.056	1.040-1.071	<0.001	1.057	1.040-1.074	<0.001
Male	0.745	0.564-0.982	0.037			0.672
Hypertension	1.215	0.902-1.638	0.201			
DM	0.807	0.574-1.135	0.218			
CHD	0.322	0.244-0.425	<0.001	0.270	0.201-0.363	<0.001
Smoking	0.655	0.486-0.884	0.006		s	0.617
Drinking	1.125	0.733-1.638	0.539			
BMI	0.102	0.994-1.074	0.102			
WBC	0.931	0.880-0.986	0.014			0.852
Neutrophil	0.940	0.885-0.99	0.042			0.938
TC	0.728	0.636-0.834	<0.001	0.813	0.704-0.939	0.005
TG	0.812	0.709-0.931	0.003			0.994
HDL-C	0.870	0.522-1.448	0.591			
LDL-C	0.724	0.615-0.852	<0.001			0.211
RDW	1.414	1.218-1.642	<0.001			0.286
Albumin	0.936	0.902-0.971	<0.001			0.176
RAR, Q1	Reference			Reference		
Q2	0.885	0.550-1.427	0.617	0.811	0.497-1.324	0.402
Q3	1.823	1.203-3.764	0.005	1.490	0.965-2.302	0.072
Q4	2.567	1.724-3.822	<0.001	2.015	1.315-3.089	0.001

After adjusting for these confounding factors, the highest quartile (Q4) of RAR was independently associated with an increased risk of AF compared with the lowest quartile (Q1) of RAR (OR:2.015, 95% CI:1.315–3.089, *P* = 0.001). Furthermore, the positive association between older age and older age remained significant (OR:1.057, 95% CI: 1.040–1.074, *P* < 0.001). In particular, the analysis revealed that CHD and LDL-C (OR:0.270, 95% CI:0.201–0.363, *P* < 0.001 and OR:0.813, 95% CI:0.704–0.939, *P* = 0.005, respectively) were markedly inversely associated with AF.

**Fig. 2:**
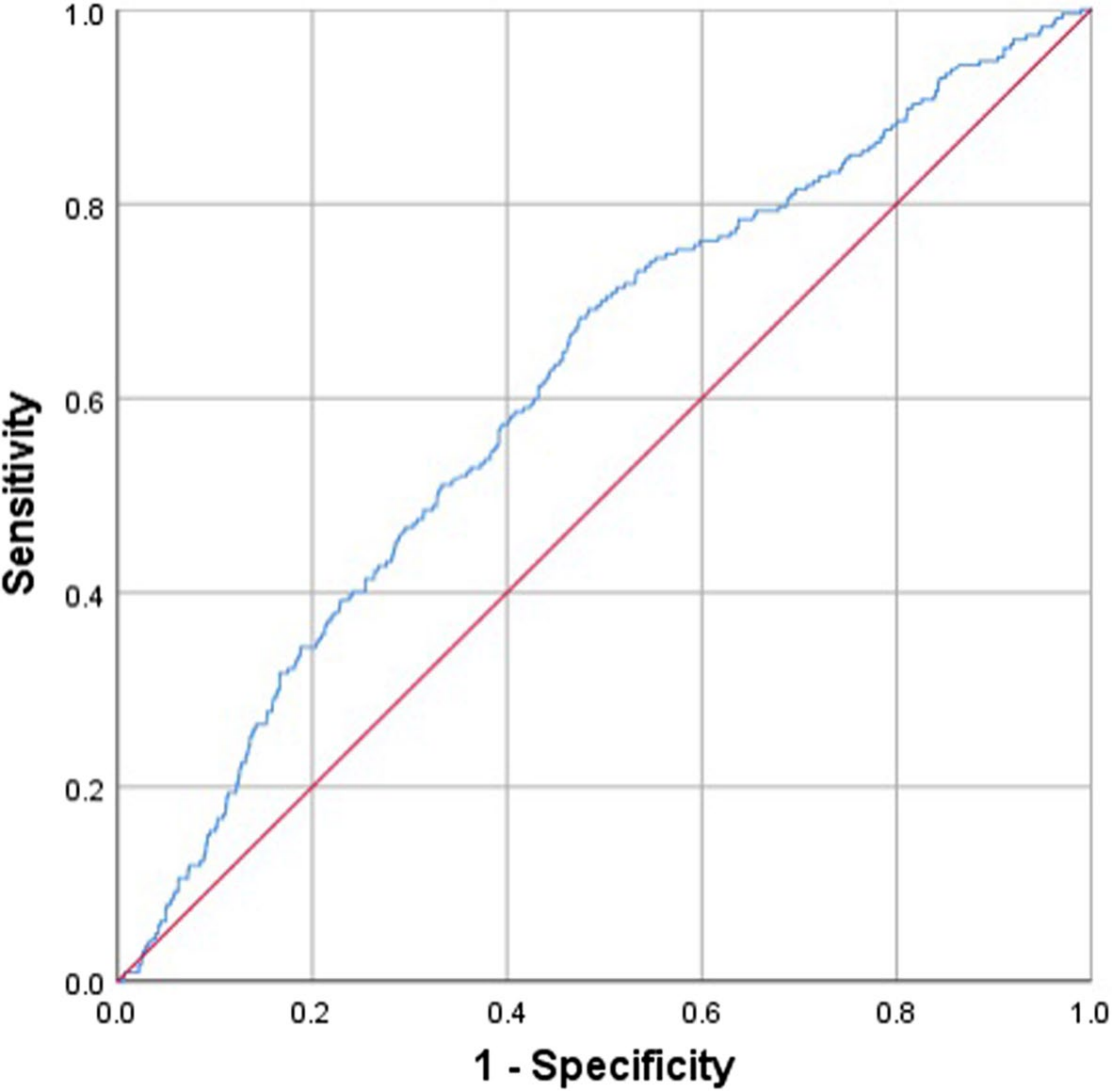
The ROC curve of the RAR to predict AF in general population. The cutoff value for the RAR was 3.21, with a sensitivity of 68.3% and a specificity of 52.3% (AUC = 0.616, 95% CI: 0.578–0.654, *P* < 0.001). ROC, receiver operating characteristics; RAR, red cell distribution width/albumin ratio; AF, atrial fibrillation; AUC, area under the receiver cure

The ROC curve analysis revealed that the RAR was a predictor of AF (AUC = 0.616, 95% CI: 0.578–0.654, *P* < 0.001). The cutoff value for the RAR was 3.21, and the sensitivity and specificity were 68.3% and 52.3% (Fig. 2).

## Discussion

To our knowledge, this is the first study focus on the relationship between the red cell distribution width to albumin ratio (RAR) and atrial fibrillation (AF) in subjects hospitalized with coronary angiography for the first time. The results suggested that elevated RAR was independently associated with AF. RAR might be a potential parameter to estimate presence of AF.

Inflammation in cardiac tissue and circulatory processes can modulates calcium homeostasis and connection, which are related with triggers and maintain of AF in the general population [22]. Moreover, studies on atrial fibrillation pathophysiology provided an update knowledge regarding inflammation and atrial fibrosis, potential as therapeutic targets [23]. Red blood cell distribution width (RDW), an index of variation of erythrocyte volume, reflects the degree of anemia traditionally. The conventional reference range of RDW is roughly comprised between 12 and 15%. Previous studies have reported that RDW could be used beyond anemia in clinics. Several pathophysiological mechanisms underling cardiovascular diseases including inflammation, oxidative stress, adrenergic stimulation and reduced iron mobilization have been reported, in which RDW play an important role [24]. A report of 2140 patients with symptomatic heart failure revealed that RDW was significantly associated with cardiovascular death or hospitalization for heart failure (HR1.17; 95%CI 1.10–1.25) [8]. Besides, it was important to note that RDW was demonstrated to be an independently indicator for sub-clinical inflammation [10, 25]. Subsequently, increasingly evidence have suggested that the RDW is a prognostic marker of AF in various clinical settings [26]. Moreover, admission levels of RDW was correlated with new-onset AF in patients after a primary percutaneous coronary intervention [27]. Similarly, in our study, the RDW levels were found to be significantly higher in patients with AF than those without AF.

Albumin, an essential protein for important physiologic effects in human plasma, was proven to be associated with chronic inflammation [28]. Albumin concentrations tend to decline in the inflammatory conditions. And linear regression analysis discovered little effect of CRP on albumin levels, although the mechanisms underlying this remain unclear [29]. A previous study comprised of 285,930 patients demonstrated that albumin level is strongly negatively correlated with the risk of cardiovascular death, as well as with the reduction of anti-inflammatory activity and oxidative stress, especially in males [30]. Based on the mechanisms, recent study conducted in 909 patients with AF in China showed that AF patients had lower levels of albumin. Lower serum albumin levels was significantly associated with AF [31]. As reported, the patients with AF recurrence after ablation had a significantly higher level of red blood cell distribution width (RDW) at baseline than those with sinus rhythm [32]. In consistent with these reports, the results in our study revealed that albumin was inversely associated with AF. Additionally, albumin was not an independent risk factor for AF after adjustment for confounding variables.

The RAR has been considered as a new and reliable parameter related with a variety of disease in clinical practice. Recent research has suggested that RAR is a better prognostic factor of aortic aneurysms rather than RDW [33]. Huang M, et al. found that RAR is significantly related with carotid plaques in patients with coronary heart disease [17]. A study of 3042 patients with sepsis and AF indicated that RAR was strongly independently associated with the risk of all-cause mortality. A linear relationship between the RAR and in-hospital mortality in patients with sepsis and AF was obtained [34].

Recently, a meta-analysis showed that higher TC levels tend to be independently attributed to an decreased risk of AF in individuals without cardiovascular disease, whereas no significant association between incidence of new-onset AF and TG, HDL-C, or LDL-C levels were found [35]. The consistent results were detected in our study. TC was verified to be inversely associated with AF progression.

Our results suggested that the RAR played an important role in the presence of AF. When we divided individuals according to the RAR levels, we found participants in the highest RAR quartile (Q4) were inclined to have higher prevalence of AF than those in the lowest quartile (Q1). Although RAR was calculated using RDW and albumin, regression analysis showed RAR was an independent risk factor for AF after adjustment of age, TC, CHD and other confounding factors. Besides, the results also proved that RAR was more credible compared to other factors such as white blood cell and neutrophil of reflecting the inflammatory status. Additionally, the RAR may be a simply measured and therapeutic target for preventing AF in clinical practice. The findings might provide valuable new insight into AF research.The exact mechanism underling the close association of RAR and AF need to explore in future studies. In conclusion, our study showed that RAR was an important indicator of the occurrence of AF. However, we have not yet seen some new therapeutic methods for this indicator, and we also hope to discover some new therapeutic drugs and methods in our future work.

Additionally, in the last decade, artificial intelligence (AI) has shown its effectiveness in CAD and AF management. The value of AI on detecting the recurrence of AF in patients underwent ablation has been explored [36]. It would be interesting to validate the AI and RAR for predicting AF in patients who underwent ablation.

There were several limitations in the present study. Firstly, this was a single-center retrospective and observational study. It was carried out in subjects hospitalized with coronary angiography. Therefore, there was unavoidably case selection bias. It was less reliable than prospective results. The findings should be further confirmed by multicenter studies in general population. We look forward to confirming them in a prospective controlled study being conducted at our center. Secondly, 12-lead electrocardiogram (ECG) or 24-h Holter might be less able to detect AF than 72-h Holter. And it could not evaluate whether RAR has a different relationship with different types of AF. Thirdly, post op AF is a known complication post CAG which may or may not be mediated by the RAR. It's our negligence that the 3 patients weren't ruled out. However, the impact on our study may be small due to the small proportion (1.32%). In our future study, new-onset atrial fibrillation after CAG will be added to exclusion criteria. Finally, nutritional status, CRP, BNP and other clinical parameters were not available in the analysis for missing too many values. So it is necessary to certify the results by large sample data in prospective study.

## Conclusions

In conclusion, results in the current study revealed that RAR was independently associated with the risk for AF in those hospitalized with coronary angiography. The RAR may be a simple and reliable clinical marker to identify patients with high risk of AF. Further studies are required to confirm the association in general population.

### Supplementary Information

**Additional file 1: Supplement Table 1.** Test methods of biochemical parameters

## Data Availability

The data used to support the findings of this study are available from the corresponding author (songyb1984@126.com) on reasonable request.

## Abbreviations

DM: Diabetes mellitus
CHD: Coronary heart disease
BMI: Body mass index;
WBC: White blood cell
TC: Total cholesterol
TG: Triglyceride
LDL-C: Low-density lipoprotein cholesterol
HDL-C: High-density lipoprotein cholesterol
RDW: Red cell distribution width
RAR: Red cell distribution width **/**albumin ratio
