# Supplementary material for: Red cell distribution width to albumin ratio is a risk factor for atrial fibrillation in subjects hospitalized with coronary angiography
Source: BMC Cardiovasc Disord. 2024 Feb 8;24:95. doi: 10.1186/s12872-024-03772-8 (PMC10854169; doi:10.1186/s12872-024-03772-8)
Supplement: Supplementary file 1 — Additional file 1: Supplement Table 1. Test methods of biochemical parameters [file 12872_2024_3772_MOESM1_ESM.docx]

**Supplement Table 1** Test methods of biochemical parameters

| Biochemical parameters | Test Methods | Measuring Range |
| --- | --- | --- |
| WBC (*10^9) | Laser flow cytometry and nucleic acid flurescence staining method | 3.50～9.50 |
| Neutrophil(*10^9) | Laser flow cytometry and nucleic acid flurescence staining method | 1.80～6.30 |
| TC (mmol/L) | Enzymatic method | 3.00～5.70 |
| TG (mmol/L) | GPO-POD method | 0～1.70 |
| LDL-C (mmol/L) | Direct method | 0～3.12 |
| HDL-C (mmol/L) | Direct method | 0.83～1.96 |
| RDW(%) | Autoanalyzer | 10.0～15.7 |
| Albumin (g/dL) | Bromocresol green method | 40.0～55.0 |

WBC, white blood cell (WBC) ;TC, total cholesterol; TG, triglyceride; LDL-C, low-density lipoprotein cholesterol; HDL-C, high-density lipoprotein cholesterol; RDW, red cell distribution width.
